# Wnt Signaling in Brain Tumors: A Challenging Therapeutic Target

**DOI:** 10.3390/biology12050729

**Published:** 2023-05-16

**Authors:** Lorenzo Manfreda, Elena Rampazzo, Luca Persano

**Affiliations:** 1 Department of Women and Children’s Health, University of Padova, Via Giustininani, 3, 35128 Padova, Italy; 2 Pediatric Research Institute, Corso Stati Uniti, 4, 35127 Padova, Italy

**Keywords:** Wnt signaling, brain development, brain cancer, Wnt targeting

## Abstract

**Simple Summary:**

Wnt signaling is one of the main evolutionarily conserved developmental pathways needed to instruct embryo formation and maintenance of organ tissues. Decades of research also clearly recognize the fundamental role of Wnt signaling during carcinogenesis. Indeed, dysregulation of Wnt pathway components has been suggested as a relevant hallmark of several neoplastic malignancies. In this review, we summarize the main molecular mechanism through which the Wnt pathway exerts its intracellular effects, with a specific focus on brain development and brain tumors, and how Wnt interacts with the surrounding brain environment. In this context, we review the latest anti-cancer therapeutic approaches employed to specifically target Wnt signaling in cancer, and their potential application in the brain tumor context. Moreover, we discuss the additional efforts that will be needed to define the real clinical impact of Wnt modulation in different types of brain tumors and even how to overcome the unsolved concerns about the potential systemic effects of such therapeutic approaches.

**Abstract:**

The involvement of Wnt signaling in normal tissue homeostasis and disease has been widely demonstrated over the last 20 years. In particular, dysregulation of Wnt pathway components has been suggested as a relevant hallmark of several neoplastic malignancies, playing a role in cancer onset, progression, and response to treatments. In this review, we summarize the current knowledge on the instructions provided by Wnt signaling during organogenesis and, particularly, brain development. Moreover, we recapitulate the most relevant mechanisms through which aberrant Wnt pathway activation may impact on brain tumorigenesis and brain tumor aggressiveness, with a particular focus on the mutual interdependency existing between Wnt signaling components and the brain tumor microenvironment. Finally, the latest anti-cancer therapeutic approaches employing the specific targeting of Wnt signaling are extensively reviewed and discussed. In conclusion, here we provide evidence that Wnt signaling, due to its pleiotropic involvement in several brain tumor features, may represent a relevant target in this context, although additional efforts will be needed to: (i) demonstrate the real clinical impact of Wnt inhibition in these tumors; (ii) overcome some still unsolved concerns about the potential systemic effects of such approaches; (iii) achieve efficient brain penetration.

## 1. Introduction

Vertebrate developing embryos are extremely complex entities. Every single embryo develops from one single cell through the instructions provided by peculiar signaling pathways able to guide cellular specification in both space and time. Most importantly, during adulthood, the same pathways are fundamental to maintain the structure and functionality of specific organs and, consequently, control the homeostasis of the entire organism. This means that during the entire life of a vertebrate organism, the disruption, imbalanced dosages, or altered activation patterns of these fundamental pathways can lead to the onset of various diseases, including cancer, which may occur at different life stages. Among the fundamental molecules responsible for the development of several organs and the maintenance of their homeostasis, the Wnt (Wingless-related MMTV integration site) family of secreted lipid-modified glycoproteins and its downstream effectors is key. In 1980, the Wnt cascade was identified as an early segmental patterning signal for the correct development of Drosophila larva [1] and a strong oncogene, able to induce breast cancer in mouse models [2]. Since then, Wnt signaling has emerged as a fundamental regulator of cell fate decisions and migration, organismal development, tissue homeostasis and, in general, cell proliferation and behavior [3,4]. Indeed, Wnt signaling serves as a crucial stimulus for the correct development of several organs and tissues, including the skin and its appendages [5], the heart [6], the liver [7], the intestinal epithelium [8], the kidney [9,10], the bones [11], as well as many others [12]. In particular, Wnt signaling plays a crucial role during different stages of brain development, from early neurogenesis to the differentiation of neural progenitors in the adult hippocampus [9,10]. Hence, abnormal regulation of these mechanisms may severely contribute to several diseases, including cancer.

It is now evident that Wnt signaling is linked with a variety of human diseases [13,14] spanning from brain pathologies [15,16] to bone [17], vascular [18], and genetic disorders [19], not forgetting cancer. As a matter of fact, a large number of previous studies indicate that Wnt signaling must be considered a crucial pathway during oncogenesis [4,20], tumor progression [21], and cancer resistance to treatments [22,23]. Moreover, based on the suggested increased Wnt signaling with aging [24], one may also correlate this observation to the substantial augmented risk of cancer onset in the older population. Familial adenomatous polyposis (FAP) is the typical example of a disease caused by dysregulation of the Wnt pathway. The causative role of Wnt signaling for FAP development was firstly identified in 1991 [25,26]. FAP patients usually develop hundreds of polyps at the level of the colon and rectum, with a high probability to progress to colorectal cancers. Wnt signaling dysregulation is also considered a hallmark of brain tumors [9], here playing a fundamental role in cell proliferation, phenotype, and modulation of their stem cell properties [27,28], as already reported for many other cancer types [21]. Based on this knowledge, the possible targeting of the Wnt/β-catenin signaling pathway has emerged as a promising therapeutic strategy for the treatment of various cancers [21]. Nevertheless, relatively little is known about the potential clinical impact of Wnt signaling inhibition in brain tumors [29].

In this review, we summarize the role played by Wnt signaling in normal brain and brain cancers. In particular, we focus on the determinant role played by Wnt during brain development and specification and in brain cancer physiology, with an important inset in the reciprocal stimulation (Wnt-dependent) occurring between cancer cells and their microenvironment. Moreover, we extensively report on and discuss the most promising pharmacological tools available for the inhibition of Wnt signaling in cancer and, possibly, in brain tumors, considering the key obstacles still present on the way to their clinical translation and administration to patients.

## 2. The Wnt Signaling Molecular Cascade

### 2.1. The Wnt Family Canonical Landscape

**Figure 1:**
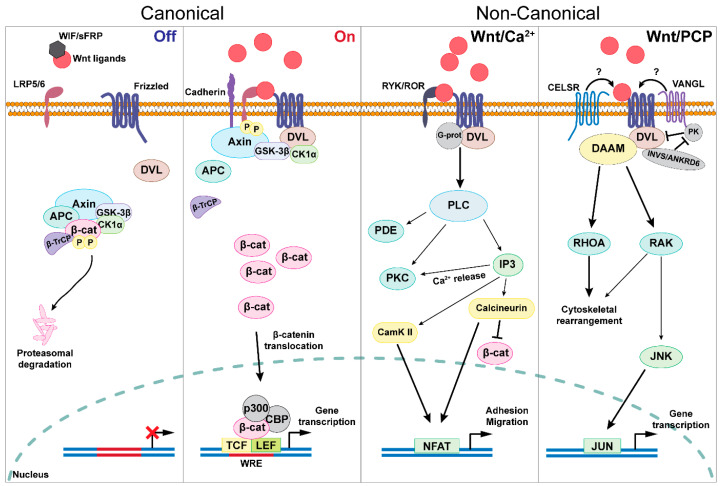
Graphic summarizing the main molecular players involved in the transduction and activation of the canonical (**left panels**) and non-canonical Wnt signaling cascades (**right panels**). In the canonical Wnt signaling, the absence of Wnt ligands allows the combination of Axin and APC to recruit GSK-3β and CK1α, which then phosphorylate β-catenin, targeting it to proteasomal degradation through β-TrCP. Upon FZD stimulation induced by Wnt ligands, DVL is recruited to the cell membrane, thus providing a site for Axin and GSK-3β to bind and phosphorylate LRP5/6, finally preventing the formation of the destruction complex. This allows the accumulation of nuclear β-catenin, which activates gene transcription through the binding with a series of transcription factors (TCF/LEF) and transcriptional co-activators (i.e., CBP and p300). The activation of downstream non-canonical Wnt pathways is almost independent on β-catenin. In the Wnt/Ca^2+^ signaling, Wnt ligands stimulate the concomitant activation of FZD receptors and RYK/ROR co-receptors leading to the activation of a PLC-dependent molecular cascade involving the activation of PDE, Inositol 1,4,5-Trisphosphate Receptor (IP3), Ca^2+^ release from the Endoplasmic Reticulum and the eventual stimulation of CamK II and Calcineurin. This signal is then transduced into a specific gene transcription pattern through the Nuclear Factor Of Activated T Cells (NFAT) transcription factor. On the other hand, in the Wnt/PCP signaling, recruited DVL proteins form a complex with DAAM which concomitantly stimulates the RHOA cascade and a RAK-dependent JNK activation, finally resulting in the recruitment of the JUN transcription factor. Created with Adobe Illustrator.

Wnt ligands comprise a family of 19 secreted hydrophobic glycoproteins that exert their function by binding to Frizzled (FZD) receptors and transmembrane low-density lipoprotein receptor-related protein 5/6 (LRP5/6) co-receptors located on the target cell surface [30] (Figure 1). The ligand–receptor interaction induces the dismantling of an intracellular destruction complex composed of the Adenomatous Polyposis Coli (APC) protein, the Axis Inhibition Protein (Axin), the Glycogen Synthase Kinase-3β (GSK-3β), and the serine/threonine (ser/thr) Casein Kinase 1α (CK1α). In particular, Wnt molecules, upon binding to FZD receptors, recruit the evolutionarily conserved protein Dishevelled (DVL) to the membrane, providing a site for Axin and GSK-3β to bind and phosphorylate LRP5/6, finally preventing β-catenin degradation. The main function of the destruction complex is to phosphorylate the β-catenin amino terminal ser/thr-rich sequence (Ser33 and 37), thus generating an E3-ubiquitin ligase β-TrCP recognition site that targets β-catenin to the proteasome for its degradation [31]. Conversely, concomitant FZD and LRP5/6 activation results in the recruitment of the β-catenin destruction complex to cadherin proteins located at the cell membrane and the formation of a membrane-associated puncta allowing β-catenin accumulation and its nuclear translocation [32] (Figure 1).

The cytoplasmic-nuclear shuttling of β-catenin is the most importantly regulated molecular mechanism which modulates the Wnt/β-catenin pathway activation. Indeed, additional tyrosine phosphorylation (Tyr142/Tyr654) of β-catenin probably dictates peculiar interactions with α-catenin and E-cadherin and results in its dissociation from cadherin-containing adherens junctions, cytoplasmic accumulation, and nuclear translocation to promote gene transcription [33]. Once released from the destruction complex, or adherens junctions, active β-catenin translocates into the nucleus to exert its function as a transcriptional co-activator by interacting with the T Cell Factor/Lymphoid Enhancer Factor (TCF/LEF) family of transcription factors [34,35]. The TCF/LEF family of genes (*TCF7*, *TCF7L2*, *TCF7L1,* and *LEF1*) encodes for specific transcription factors (TCF1, TCF4, TCF3, and LEF1, respectively) that bind to DNA through a SRY-Box Transcription Factor (SOX)-like high mobility group domain, which recognizes a specific DNA consensus around the core “CTTTG” sequence, known as the Wnt Response Element (WRE) [36] (Figure 1). Recent research has demonstrated that more than 85% of the β-catenin-dependent transcriptional effects directly depend on TCF/LEF transcription factors and that the genome-wide physical association of β-catenin with its specific consensus sequences is severely affected when TCF/LEF are mutated or knocked-down [37]. Despite the consolidated and pivotal role of the β-catenin-TCF/LEF complex, recent findings highlight the existence of other branches within the canonical Wnt/β-catenin signaling pathway that act independently from TCF/LEF transcription factors. In particular, this has been demonstrated in the physical and functional interaction between β-catenin and the Hypoxia-Inducible Factor-1α (HIF-1α) [38], the basic helix-loop-helix (bHLH) transcription factor essential for muscle differentiation MyoD [39], various SOX proteins [40], and many members of the Forkhead Box O (FOXO) family of transcription factors [41]. This intricate, and potentially redundant, set of β-catenin-containing protein complexes, able to interact with DNA and regulate the expression of target genes, confirms the involvement of Wnt signaling in several essential biological processes controlling cell behavior and tissue integrity. In particular, these have been described to heavily affect embryonic development [42], stem-cell maintenance and differentiation [12], bone regeneration [43], but also to support the onset of many diseases such as cancer [44], diabetes [45], and oxidative stress and inflammation [46,47].

### 2.2. The Non-Canonical Wnt Signaling

In addition to the previously described canonical signaling, the Wnt pathway may also take advantage of alternative non-canonical molecular mechanisms. The term non-canonical pathway refers to the Wnt-dependent but β-catenin-independent signaling pathways. The two main and well-characterized non-canonical Wnt pathways are the planar cell polarity (PCP) and the Wnt-Calcium (Wnt/Ca^2+^) (Figure 1).

Wnt/PCP signaling is an evolutionarily conserved pathway, both in vertebrates and invertebrates, whose function is to control polarized cell behavior, a process that involves the asymmetric distribution of a set of core proteins within the cell and the subsequent cell polarization across the tissue plane [48,49]. Genetic studies performed across different stages of Drosophila development identified a group of core PCP proteins: Frizzled (Fz), Van Gogh (Vang), Dishevelled (Dsh), Prickle (Pk), Diego (Dgo), and Flamingo (Fmi), which control the orientation of ommatidial clusters in the eye disc and of the bristles and hairs on the fly body [50]. In vertebrates, such as Zebrafish, PCP is fundamental for the convergent extension movement process and serves as a key determinant of the elongation of the anterior–posterior body axis [51]. In mammals, the PCP complex, composed of Frizzled receptors (FZD1-10), Van Gogh-like proteins (VANGL1-2), Dishevelled transducers (DVL), Prickle Planar Cell Polarity Protein (PRICKLE1-3) nuclear receptors, Inversin/Diversin proteins (INVS/ANKRD6), and the Cadherin EGF LAG seven-pass G-type receptors (CELSR1-3), has been shown to control a diverse array of cellular, developmental, and physiological processes whose disruption determines a great variety of developmental defects and prenatal abnormalities [52,53]. The DVL-dependent Wnt/PCP signals are transduced to the Ras Homolog Family Member A (RHOA) signaling cascade through the Formin proteins Dishevelled Associated Activator of Morphogenesis (DAAM1-2) [54,55,56,57] (Figure 1). Furthermore, the altered activation of PCP has been implicated in the progression of various cancers. Indeed, the PCP pathway is extremely sensitive not only to the expression levels of the above-mentioned core proteins, but also to how the core proteins interact both at the intracellular and intercellular level, thus inducing the correct establishment of the PCP cellular asymmetry or a randomized polarity [58]. In this context, a better understanding of how PCP signaling is transduced and finely molecularly regulated is, therefore, essential to harness this signaling pathway for therapeutic purposes.

In Wnt/Ca^2+^ signaling, the Wnt-FZD binding and, in particular, the binding of the Wnt-5a ligand to the FZD5 receptor and the Receptor Tyrosine Kinase Like Orphan Receptor (ROR1/2) family of co-receptors, leads to the activation of the DVL family of transducers (Figure 1). Through these intracellular effectors, FZD receptors activate the heterotrimeric GTP-Binding Proteins (GTP-BP), leading to the activation of Calcium/Calmodulin-dependent Kinase II (CamK II), Protein Kinase C (PKC), and Phospholipase C (PLC), together with a release of Ca^2+^ from the endoplasmic reticulum [54,57,59,60]. In particular, by recruiting different DVL transducers, FZD receptors can activate phosphodiesterase (PDE) and the smaller GTP-binding proteins, such as RHO and Cell Division Cycle 42 (CDC42), involved in cell cycle and cell migration [61]. The Wnt/Ca^2+^ pathway plays a fundamental role in early vertebrate development by regulating blastula cell fate, orchestrating morphogenetic movement during gastrulation [62,63], and finely tuning organogenesis, particularly in the nervous system, the hematopoietic compartment, and the cardiocirculatory system, together with many other ventral organs [64,65]. A pivotal and controversial role of the Wnt/Ca^2+^ pathway is still under investigation in the cancer context where its activation can act both as a proto-oncogene or a tumor suppressor, depending on the cell type and the specific expression of pathway receptors [66]. As an example, Wnt-5a stimulation is sufficient to induce melanoma cell metastasis by inducing epithelial to mesenchymal transition (EMT) through the PKC/Ca^2+^ cascade [67,68]. On the contrary, Wnt-5a was reported to act as a tumor suppressor in neuroblastoma [69] and colon cancer [70].

## 3. Wnt Signaling in Central Nervous System Development

**Figure 2:**
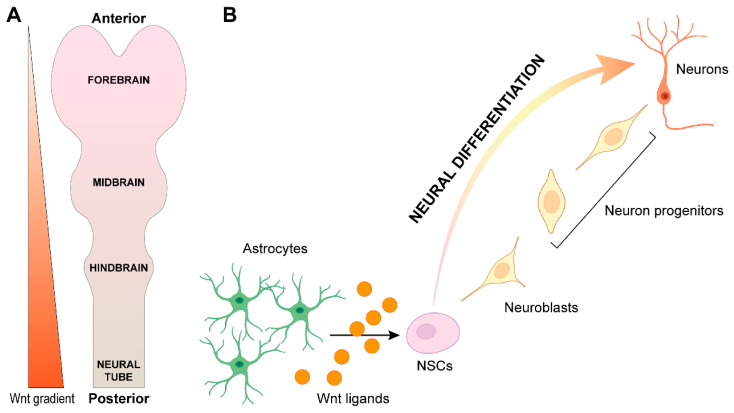
(**A**) Graphic representing the developing neural tube whose anteroposterior patterning is dictated by a Wnt signaling activation gradient. (**B**) Schematic representation of the NSC niche in which Wnt ligands, released by astrocytes, induce their neuronal specification. Created with Adobe Illustrator. A few of the icons were sourced from BioRender.com (accessed on 26 April 2023).

In 2001, Kiecker and Niehrs demonstrated that an activity gradient of Wnt/β-catenin signaling acts as a transforming morphogen to pattern the Central Nervous System (CNS) in Xenopus. In particular, they found that the Wnt signaling gradient is higher in the posterior and lower in the anterior Xenopus body axis, thus properly specifying the anterior–posterior polarity of the neural plate during development [71] (Figure 2A). As soon as the neural plate is specified, the invagination process starts to generate the neural tube. The already mentioned Wnt/PCP non-canonical signaling has been demonstrated to play a key role in neural tube closure. Indeed, mouse-based genetic studies unraveled that specific mutations in the Wnt/PCP core proteins such as FZD4, Wnt-11, CELSRs, LRP6, VANGL2, and PRICKLE-1 result in neural tube defects (NTDs) due to failure of the neural tube closure [72]. In addition, the Wnt/β-catenin canonical pathway and its finely tuned regulations also contribute to neural tube formation. In this context, the LRP6-dependent Wnt/β-catenin signaling allows the posterior neuropore closure and elongation in both mice [73] and humans [73], by means of a β-catenin-mediated activation of the transcription factors Paired Box 3 (PAX3) and Caudal Type Homeobox 2 (CDX2) [74]. Based on this knowledge, the manipulation of both the canonical and non-canonical Wnt signaling activities could provide a relevant therapeutic target for NTDs.

The neural tube is made up of pluripotent precursor cells that proliferate, commit to a specific cell fate, and then migrate to their final destination to arrange the different layers of the CNS, including neuronal ganglia and nuclei, and the cerebral cortex. The Wnt/β-catenin pathway has been associated with both proliferation and specification of neural stem cells (NSCs) during CNS development, in cooperation with other vestigial developmental pathways such as Notch, Sonic Hedgehog (SHH), Bone morphogenetic protein (BMPs) and Fibroblast Growth Factor (FGF) signaling. Indeed, the dorsal–ventral/rostral–caudal gradients of these morphogens and their relative receptors, together with specific cell–cell contacts between NSCs, finely specify the differentiated cell types that compose the nervous system, including mature neurons, astrocytes, and oligodendrocytes [75]. The Wnt/β-catenin pathway maintains the stemness of NSCs by positively or negatively modulating the activity of the BMP pathway during embryonic development, depending on the peculiar microenvironment NSCs are exposed to during specific developmental stages [76,77]. Once the brain is fully developed, the pivotal regulatory role that Wnt signaling still maintains during adult neurogenesis has been demonstrated, by acting as a neuronal pro-differentiation signal. Indeed, several Wnt family members, including Wnt-3, have shown to be expressed by adult hippocampal astrocytes, thus stimulating neuroblast proliferation and instructing adult hippocampal neural progenitors to acquire a neuronal fate [78] (Figure 2B). In particular, Kuwabara and co-workers demonstrated that Neuronal Differentiation 1 (NeuroD1), a pro-neurogenic bHLH transcription factor, is a downstream effector of Wnt signaling, needed to induce an efficient neuronal differentiation [10]. In addition, the β-catenin/TCF complex directly induces the expression of Neurogenin 1 (*NGN1*), which participates in stimulating cortical neuronal differentiation [79].

Besides substantial evidence for the role of Wnt/β-catenin in controlling neurogenesis through the promotion of neuronal differentiation, some studies have demonstrated that this signaling can also stimulate neural progenitor cell proliferation, since the β-catenin-dependent signal induces the expansion of proliferating precursors in the sub-ventricular zone [80,81]. Therefore, it seems that Wnt/β-catenin may affect both proliferation and differentiation of neural precursors in the CNS, depending on the concomitant activation of other signaling cascades in restricted brain loci and developmental stages [75].

As suggested by the above considerations, it is clear that any dysregulation occurring at the level of both canonical and non-canonical Wnt signaling components may severely affect the fine equilibrium existing in the cellular composition of the CNS, eventually contributing to the onset, progression, and peculiar behavior of different brain tumors. Accordingly, in the following sections, the major known roles played by Wnt signaling in a series of brain cancers are described, thus providing the rationale for considering Wnt signaling as a relevant therapeutic target against brain malignancies.

## 4. Wnt Signaling in Brain Tumors

### 4.1. Unraveling the Complexity of Wnt Signaling in Glioblastoma

The involvement of the Wnt signaling pathway in brain tumors has been extensively reported, with its functionality found to be largely contingent upon the tumor subtype [9,82,83]. This pathway has been subjected to intensive investigation in the context of Glioblastoma Multiforme (GBM) [22], recognized as one of the most lethal and aggressive brain tumors [84]. Indeed, despite the implementation of highly aggressive therapeutic approaches, including surgical resection, radiotherapy, and chemotherapy, GBM patients display a median progression-free survival of 12–15 months, with only 3–5% of individuals surviving beyond 3 years [85,86,87]. Currently, recurrent GBM is nearly untreatable, as no targeted therapies have been authorized for its effective eradication. Accordingly, the low survival rate of GBM patients is primarily attributable to disease recurrence, which arises in nearly all patients after completion of the available standard treatments, due to their intrinsic resistance to any additional chemotherapy and radiotherapy cycle [88].

The role of Wnt signaling in the onset and progression of GBM has been extensively studied and characterized, resulting in complex and sometimes contradictory evidence [89,90,91,92,93]. According to recent studies, the contribution of the Wnt pathway to GBM features has been demonstrated to be highly heterogeneous, depending, to different extents, on the microenvironment, the experimental conditions, and the specific experimental models employed. As a result, it remains unclear whether Wnt has a positive or negative impact on GBM development, progression, and aggressiveness [89,94,95]. The clarification of this dichotomy is not obvious and requires careful consideration of multiple microenvironmental factors, starting from the pivotal role played by the intra-tumoral oxygen availability [96].

#### 4.1.1. The Role of Microenvironmental Oxygen

As discussed in previous sections, Wnt pathway activation requires the participation of several, and sometimes redundant, molecular transducers, co-activators, repressors, transcription factors, etc., thus making its study very complex and challenging. In GBM, it is well-known that the binding of Wnt ligands to FZD receptors activates the canonical β-catenin-dependent signaling pathway, which then promotes the expression of target genes through the formation of a large molecular complex together with TCF/LEF transcription factors and CREB Binding Protein (CBP)/p300 transcriptional co-factors [3,28,30,97,98,99] (Figure 1). In normoxic conditions (i.e., environmental 20% oxygen), it has been demonstrated that canonical Wnt signaling activation enhances the expression of some EMT activators in GBM cells, including Zinc Finger E-Box Binding Homeobox 1 (ZEB1), Twist Family bHLH Transcription Factor 1 (TWIST1), and Snail Family Transcriptional Repressor 2 (SLUG), thus enhancing their migratory properties in vitro. In the same conditions, it has been reported that increased Wnt activation, dependent on several mechanisms, including both genetic and epigenetic factors, sustains Glioma Stem Cell (GSC) maintenance and function [99,100,101,102,103]. For example, amplification/gain of PLAG1 Like Zinc Finger 2 (*PLAGL2*) expression has been linked to the upregulation of FZD2-9 receptors, thus promoting Wnt pathway activation and contributing to GSC self-renewal and maintenance [104]. Moreover, Wnt-regulated Forkhead Box M1 (FOXM1) also potentiates GBM cell stemness by directly binding the promoter and thus activating the expression of the NSC transcription factor SOX2 [105]. 

Further confirming a prominent Wnt signaling activation in GBM cells, through large-scale whole genome approaches it has been demonstrated that they exhibit epigenetic-dependent decreased expression of several Wnt pathway inhibitors, such as Wnt Inhibitory Factor 1 (WIF1), Dickkopf inhibitors (DKKs), and Secreted Frizzled Related Protein 1 (SFRP1) [29,101,106,107,108]. Intriguingly, authors have shown that these tumor suppressors are epigenetically silenced by histone modification and DNA methylation in their promoter region and that histone deacetylase (HDAC) inhibition, but not azacytidine treatment, is sufficient to restore the expression of all three genes, with a clear impact on cell proliferation [100,109,110]. We should also emphasize that activated Wnt/β-catenin signaling has been associated with increased activity of O6-Methylguanine-DNA Methyltransferase (MGMT). Importantly, this enzyme serves as an efficient DNA repair mechanism for GBM cells, shielding them against the mutagenic impact of the alkylating agent temozolomide, the gold standard chemotherapeutic drug administered to patients during adjuvant therapy [23,111,112]. Accordingly, increased levels of the Wnt signaling inhibitor DKK-1 have been demonstrated to enhance the sensitivity of GBM cells to chemotherapy [113]. Finally, from a more translational point of view, Wnt/β-catenin activation has been generally associated with a decreased survival of GBM patients [114], underlining the importance of additional studies aimed at defining the precise contribution of canonical Wnt signaling components to GBM onset, progression, and relapse.

**Figure 3:**
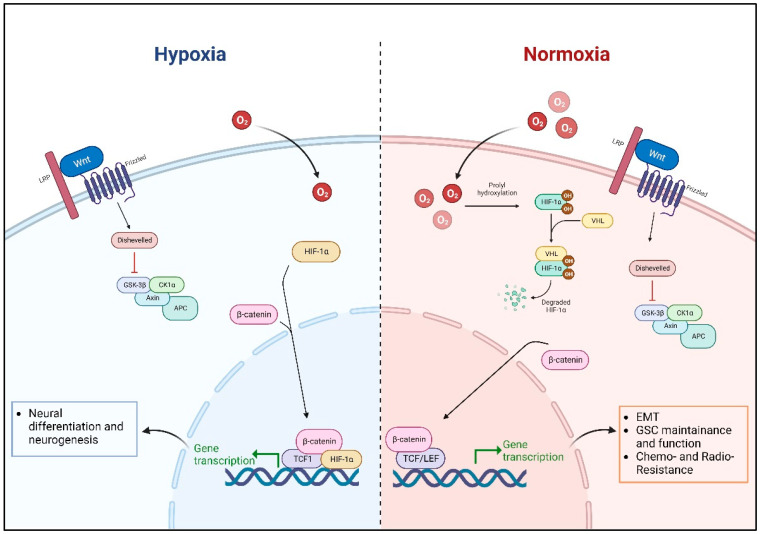
Graphic summarizing the differential response of Wnt signaling activation in GBM cells upon modulation of oxygen (O_2_) tension. In normoxic conditions, Wnt signaling activation promotes the translocation of β-catenin into the nucleus, where it associates with TCF/LEF transcription factors to promote gene transcription, resulting in the maintenance and function of GSCs, EMT, and resistance to chemotherapy and radiation. In hypoxia, however, HIF-1α translocates into the nucleus and interacts with TCF1 and β-catenin, promoting neuronal differentiation and neurogenesis. Created with BioRender.com (accessed on 3 May 2023).

Data presented so far seem to delineate a quite shared consensus on the fundamental role played by the Wnt pathway in GBM, by promoting cell motility and invasion through increased EMT, sustaining the growth and maintenance of GSCs, and contributing to intrinsic chemo- and radio-resistance [115]. On the other hand, multiple studies performed in tightly controlled microenvironmental conditions (i.e., hypoxia) suggest that Wnt signaling activation may exert opposing effects in GBM. Indeed, several normal and pathological tissues, including the brain and GBM, are known to be exposed to reduced oxygen tensions [116,117,118,119,120]. In the case of GBM, these appear to act as a fundamental modulator of Wnt pathway-dependent effects. It has been demonstrated that Wnt pathway activation in the normal brain [121] or GBM may promote different cellular functions based on oxygen availability, with conflicting effects observed upon Wnt signaling stimulation in hypoxic or normoxic environments. In particular, we previously demonstrated that Wnt pathway activation under hypoxic conditions (depending on the presence of a functional HIF-1α protein) promotes a strong differentiation of GSC toward a neuronal phenotype through a NUMB Endocytic Adaptor (NUMB) protein-dependent Notch signaling impairment [122,123]. Based on this evidence, we recently proposed a molecular mechanism that accounts for a potential dual role of Wnt in either inducing differentiation or maintaining GSCs, depending on intra-tumoral hypoxia and TCF proteins availability. Indeed, members of the TCF/LEF family of transcription factors are extremely heterogeneous in structure and function, with their intracellular assortment able to potentially influence the behavior of neural cells, but also GBM cells, during stem cell maintenance and differentiation [124]. Accordingly, we demonstrated that the Wnt signaling-induced formation of a HIF-1α/TCF1/β-catenin complex activates a potent pro-neuronal transcriptional program in GBM cells, which is counteracted, in normoxia, by the accumulation of high-molecular-weight TCF4 isoforms, which act as transcriptional repressors and prevent the complex binding to DNA [125] (Figure 3).

Since we provided examples that Wnt signaling may serve both as a pro-cancerous and a pro-differentiation stimulus in GBM, a better comprehension of the complex molecular interactions contributing to Wnt signaling modulation is a mandatory goal to be achieved soon. This will allow us to increase our molecular knowledge of GBM biology and even identify relevant intracellular signaling nodes with potential therapeutic impact. Nevertheless, despite the reported dichotomous effect of Wnt signaling activation in GBM tumors, several studies still consider Wnt inhibition as a promising therapeutic strategy in these tumors.

#### 4.1.2. Wnt Signaling-Dependent Remodeling of GBM Microenvironment

**Figure 4:**
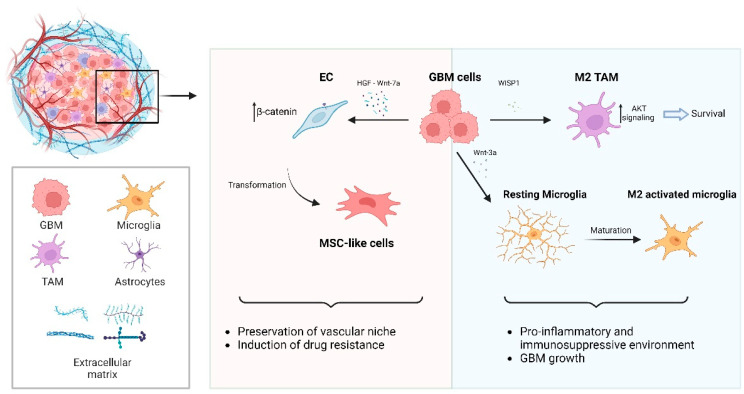
Graphic displaying the referenced Wnt-dependent remodeling of the microenvironment in GBM. GBM cells secrete HGF, which induces EC transformation into MSC-like cells by accumulating nuclear β-catenin. This transformation enhances the survival of both GBM cells and ECs upon chemotherapy. In addition, tumor cells release WISP1, which promotes the survival of M2 TAMs by activating AKT signaling. Furthermore, Wnt-3a induces the maturation of microglia into M2-activated microglia, which contributes to a pro-inflammatory and immunosuppressive environment that supports the growth of GBM cells. Together, these mechanisms allow GBM cells to shape their microenvironment in a way that sustains their growth and survival. Created with BioRender.com (accessed on 3 May 2023).

In the previous section, we described the intimate molecular dependency between oxygen availability and the modulation of Wnt pathway-dependent functions. However, it is important to recognize that this relationship is not one-sided, but rather bidirectional. In this paragraph, we aim to summarize the existing knowledge on how the Wnt pathway can affect tumor microenvironment (TME), with a focus on how GBM tumors, through activation of the Wnt pathway, can manipulate neighboring cell behavior and growth. GBM tumors evolve within an intricate and interdependent microcosm of diverse cellular components, such as immune cells, normal astrocytes, and blood vessels, as well as a dense extracellular matrix [126,127]. The TME composition has been shown to guide the fate and the phenotype of GBM cells, by sustaining proliferation, angiogenesis, invasion, and resistance to treatments [128,129,130,131,132,133,134,135]. Intriguingly, the Wnt pathway may be involved in some of these processes. A recent study elegantly demonstrated that GBM cells can stimulate endothelial cells (ECs) to transdifferentiate into mesenchymal stem-like cells, thus sustaining chemotherapy resistance [136]. This appears to be mediated, at least in part, by a Hepatocyte Growth Factor (HGF)/MET Proto-Oncogene Receptor Tyrosine Kinase (MET) signaling-dependent Wnt pathway activation, nuclear β-catenin accumulation, and Multidrug Resistance-associated Protein-1 (*MRP-1*) expression, eventually promoting EC stemness and chemoresistance (Figure 4). Accordingly, the pharmacological inhibition of Wnt signaling was shown to decrease MRP-1 expression in ECs and improve mouse survival, when combined with TMZ treatment. Intriguingly, this molecular loop not only influences EC chemoresistance, but also the response of GBM cells to chemotherapy [136]. These findings align with the observation that GBM cell-released Wnt-7a can stimulate vessel co-option, further reducing therapy response [133].

Recently, the Wnt signaling pathway has also emerged as a key player in the regulation of immune cell behavior in GBM tumors. The composition of immune cells infiltrating the GBM TME is highly variable during tumor progression. These include tumor-associated macrophages (TAMs), neutrophils, dendritic cells, plasmacytoid cells, lymphocytes, natural killer cells, mast cells, and a significant amount of microglia [137,138]. In recent years, accumulating evidence suggests that the Wnt pathway strongly influences tumor-associated microglia in GBM. Several studies have demonstrated that co-culture of GBM and microglia, or stimulation of microglial cells by GBM cell-conditioned medium, leads to Wnt pathway activation [139,140]. This enhances microglial cell proliferation and their maturation towards an M2 phenotype, significantly contributing to the onset of a pro-inflammatory and immunosuppressive environment, with a negative impact on patient prognosis. These effects seem to be mediated by the secretion of Wnt-3a [140] or other secreted proteins, such as Wnt-1 Induced Secreted Protein 1 (WISP1), from GBM cells [141] (Figure 4). In this context, Tao et al. have provided a clear explanation of the dual role played by WISP1 in promoting the growth of GBM cells, through both autocrine and paracrine mechanisms. Specifically, WISP1 supports the maintenance of GSCs through autocrine signaling by interacting with Integrin α6β1, which activates the AKT pathway, eventually promoting cell survival. Moreover, the paracrine interaction between WISP1 and tumor-associated macrophages (TAMs) enhances their maintenance and function, which in turn promotes GBM growth by sustaining the pro-inflammatory and immunosuppressive tumor niche. To support their hypothesis, the authors demonstrated that inhibiting the WISP1 signaling pathway, or targeting its upstream regulators, disrupts GSC maintenance, inhibits TAM survival, and effectively suppresses GBM growth. These findings suggest that targeting WISP1 signaling may be a potential therapeutic strategy for GBM treatment. [141,142].

### 4.2. Medulloblastoma: Focus on the Wnt Subgroup

Cerebellar medulloblastoma (MB) is a highly malignant (grade IV) and invasive brain tumor with a preferential manifestation in the pediatric age. MBs are classified into four molecular groups: Wnt-activated, Shh-activated, group 3, and group 4 MBs [143]. These subgroups were established from cluster analyses performed on genome, transcriptome, methylome, and microRNA profiling of MB tumor samples [144,145,146], consistently displaying a significant correlation with clinical data, histopathological features, and patient survival in both children and adults [144,147,148]. 

The best characterized is the Wnt subgroup, due to its very good long-term survival of patients, exceeding 90% [149]. Several molecular alterations have been described for this neoplasm, including isochromosome 17q (50% of cases), monosomy of chromosome 6, and Tumor Protein P53 (*TP53*), Patched 1 (*PTCH1*), and β-catenin (*CTNNB1*) gene mutations [150]. It is noteworthy that MB occurs in a considerable proportion (40%) of FAP patients [151], but *APC* mutations have also been found in a fraction (4.3%) of sporadic MB [152]. Moreover, *CTNNB1* mutations, considered the main hallmark of Wnt-driven MB, are found in 86% of patients, clearly suggesting the molecular dependency of Wnt MB onset and progression on the canonical Wnt/β-catenin signaling [153].

In 2012, Gibson et al. generated a mouse model of Wnt-driven MB (brain lipid-binding protein (Blbp)-Cre; Ctnnb1^+/lox(Ex3)^; Trp53^flx/flx^) in which a conditional stabilized allele of *Ctnnb1* is targeted to neural progenitor cells of the lower rhombic lip [154]. These mice, expressing the activated *Ctnnb1* transgene in a *Trp53*-deleted cellular context, develop classical MB tumors [155], clearly correlated with the human Wnt MB counterparts. Moreover, for the first time, they demonstrated that Wnt MB tumors very likely arise from the dorsal brainstem. In 2012, this model was further tuned by the addition of the PI3K catalytic-α polypeptide mutant allele (*Pik3ca*^E545K^), previously identified in human MB [156], thus generating Blbp-Cre; Ctnnb1^+/lox(Ex3)^; Trp53^+/flx^; Pik3ca^E545K^ mice, which develop Wnt-like MB tumors with 100% penetrance within 3 months [156]. These tools are fundamental to understanding the deep molecular mechanism underneath Wnt MB insurgence and to better specify the Wnt MB cell of origin. Moreover, from a therapeutic point of view, these could be considered invaluable tools for the in vivo study of possible chemotherapy de-escalation protocols, based on the extremely favorable prognosis displayed by these patients.

### 4.3. Other Brain Tumors

There is limited understanding of the impact of Wnt signaling on the development, growth, and aggressiveness of other brain tumors. However, previous research has explored the involvement of the Wnt pathway in meningiomas and pituitary adenomas.

Meningiomas (MG) are usually considered benign tumors originating at the level of brain meninges, more precisely from meningothelial arachnoid cells [157]. They are the most common CNS tumors with a generally good prognosis, depending on the localization and extension of the mass. To date, there is no approved drug therapy for the treatment of MG [158], even for the 20% of MG cases for which surgery is not curative. Several studies have shown that the Wnt pathway may play a significant role in MG, with genes involved in this signaling being differentially expressed between non-malignant leptomeningeal cells and malignant MGs [159]. In addition, Wrobel et al. reported the overexpression of various Wnt pathway-correlated genes, such as *CTNNB1*, Cyclin-Dependent Kinase 5 Regulatory Subunit 1 (*CDK5R1*), Cyclin D1 (*CCND1*), and Ectodermal-Neural Cortex 1 (*ENC1*) in atypical and anaplastic MGs, relative to benign tumors [160]. Similarly, the downregulation of specific micro-RNAs, predicted to target Wnt-related genes, has also been demonstrated in anaplastic compared to benign MGs [161]. Supporting these findings, various studies have demonstrated a heterogeneous expression of certain Wnt signaling components such as *TCF3*, *SFRP3*, *SFRP1*, Cadherin 1 (*CDH1*), and *FZD7*, when comparing atypical, anaplastic, and benign MGs [162,163]. Finally, recent studies have shown that the activation of the Wnt-FOXM1 axis, at both the genomic and epigenomic level, is associated with poor prognosis, highly mitotic phenotype, and increased aggressiveness in the most severe cases of MG [164,165].

Pituitary adenomas (PA), which are typically benign tumors originating from the pituitary gland [166], are the subject of an ongoing debate regarding the role of Wnt in contributing to their development. On one hand, it has been observed that increased Wnt signaling in pituitary progenitor/stem cells can lead to the formation of PAs in both humans and mice [167]. Additionally, overexpression of *WNT4* has been linked to the over-activation of β-catenin-dependent and independent pathways, eventually increasing PA invasiveness [168,169,170]. On the other hand, some studies have not confirmed this difference in the expression of Wnt-related genes. Nonetheless, recent research has shown that decreased expression of *SFRP2* is associated with the development of corticotrophic adenomas [171], while overexpression of Solute Carrier Family 20 Member 1 (*SLC20A1*), which may be linked to the Wnt pathway, has been associated with larger tumor size, invasive behavior, and tumor recurrence in somatotroph adenomas [172].

## 5. Wnt Signaling as a Therapeutic Target: Achievements and Challenges

In this section, we will try to provide a comprehensive description of the most promising approaches developed in the last years to achieve a therapeutic anti-cancer targeting of the Wnt/β-catenin pathway, and some examples of their application to treat brain tumors. In this context, it is worth investigating the potential employment of Wnt inhibition on certain brain cancers, particularly MB. Indeed, although aberrant Wnt signaling activation is the recognized oncogenic driver of Wnt MBs [145], its overexpression eventually results in a vascular dysfunction-induced weaker blood–brain barrier. This seems not to ease cancer cell invasion but may rather contribute to the relatively good outcome of these patients due to a chemo-sensitization mechanism and even a less invasive disease [173,174,175]. As a consequence, the inhibition of the Wnt pathway in these MB tumors may reduce chemotherapy penetration, with no trials initiated for Wnt targeting, but rather focused on treatment de-escalation [174]. Furthermore, inhibition of Wnt signaling in MB tumors belonging to other subgroups has been only proposed for very rare relapses occurring in good prognosis patients, with restricted verification in preclinical models [176]. Finally, in contrast to the above-described role of Wnt signaling over-activation as a driver of tumorigenesis, some studies have suggested that, in peculiar contexts, β-catenin overexpression may even result in anti-tumoral effects in MBs and GBM, by reducing proliferation and self-renewal of cells and prolonging the survival of MB murine models [122,125,177,178,179]. For all these reasons, the following applications of therapeutic Wnt signaling inhibition against brain tumors are nearly limited to gliomas, particularly GBM.

In general, Wnt signaling inhibitors may be classified into four quite broad groups: (1) non-steroidal anti-inflammatory drugs (NSAIDs), (2) vitamins, natural compounds, and their derivatives, (3) small molecule chemical inhibitors (with a direct or even indirect effect on Wnt signaling), and (4) antibodies against Wnt pathway components (Table 1).

### 5.1. NSAIDs

NSAIDs are drugs of common use and some of them, including aspirin, indomethacin, sulindac, and celecoxib, have been proposed for the treatment of different types of cancer. Besides their anti-inflammatory properties that may represent by themself an adjuvant strategy during cancer prevention and therapy [106], NSAIDs have been reported for a long time to specifically reduce Wnt/β-catenin activation in human cancer cells and animal models [181]. NSAIDs attenuate the synthesis of prostaglandin by inhibiting cyclooxygenase enzymes (COX-1 and COX-2), demonstrating a prominent anti-inflammatory effect and a relatively good brain distribution [269]. Interestingly, one of their proposed mechanisms of Wnt signaling interference is a COX-dependent modulation of prostaglandin levels, which in turn can affect β-catenin stability [28]. However, NSAIDs have also shown anti-cancer effects in COX activity-lacking cancer cells, thus revealing a COX-independent mechanism of action and a potential direct effect on peculiar Wnt signaling components [270]. In this context, aspirin and indomethacin have been reported to hamper β-catenin/TCF complex transcriptional activity [181], with a suggested onco-preventive action, through inhibiting the Wnt/β-catenin signaling pathway [180,182,184]. Aspirin treatment also reduced proliferation, invasiveness, and Wnt-signaling target genes in GBM cell lines [183]. A similar effect, including the reduction of nuclear β-catenin accumulation, was demonstrated for sulindac, when used to treat colon cancer [185,186]. Moreover, sulindac treatment was shown to induce differentiation of GSCs, although without suggesting a specific mechanism of action [187]. In line with these studies, the selective COX-2 inhibitor celecoxib was shown to inhibit Wnt signaling in colon cancer cell lines, by inducing the degradation of TCFs and hampering the expression of Wnt signaling target genes, independently from COX-2 activity [46,188,190]. In GBM, both diclofenac and celecoxib similarly inhibited cell proliferation and migration [189], supporting a phase II clinical trial for their combination with temozolomide, which unfortunately did not establish any benefit for patients [271].

From a clinical point of view, celecoxib is the only NSAID approved by the Food and Drug Administration (FDA) for the reduction in the number of colorectal polyps in FAP patients. However, despite apparent effectiveness, reports of COX-2 inhibitor-dependent cardiotoxicity now limit their use in FAP patients [272], supporting their more recent market withdrawal by the European Medicines Agency.

### 5.2. Vitamins, Natural Compounds, and Derivatives

Natural compounds are a major source of drugs with anti-cancer properties. Indeed, at least one-third of drugs FDA-approved for the treatment of cancer are composed of natural products or their direct derivatives [273]. Vitamins and natural compounds can influence very different molecular processes of Wnt signaling, from modulating the abundance of pathway components and their regulators to affecting Wnt-dependent transcriptional activity. In particular, although the mechanism by which Wnt/β-catenin signaling is inhibited by certain vitamins is not completely explained, it has been reported that vitamin-activated nuclear receptors may compete with TCFs for the binding with β-catenin, eventually hampering its transcriptional activity [274,275]. Moreover, vitamin A-derived retinoids, besides their recognized pro-differentiating effects in various cancers, have been suggested to interfere with β-catenin intracellular localization [276] and to induce Wnt signaling inhibitors such as Disabled-2 (Dab2) and Axin [191]. Similarly, vitamin D was shown to reduce Wnt signaling activity through DKK-1 and 4 induction in colon cancer [192]. In this context, morphogens such as Differentiation-Inducing Factors (DIFs) potently inhibit cancer cell proliferation, with DIF-1 and 3 being demonstrated to suppress Cyclin D1 expression by activating GSK-3β [193,194].

In addition to the above-mentioned pro-differentiating compounds, several other drugs of natural origin have shown promising Wnt signaling inhibition-dependent, anti-cancer (including GBM) effects, although their proposed mechanisms of action would benefit from further clarification, due to lack of specificity. Nevertheless, curcumin [195,197] and shikonin [196] displayed inhibitory effects on β-catenin activation. In glioma, trichostatin [199] and diallyl trisulfide [199] modulated the expression of Wnt signaling components LGR5 and LRP6, respectively. Both the extracts of the root *Rhodiola crenulata* [201] and resveratrol [200] have been shown to affect β-catenin localization. Quercetin disrupted β-catenin/TCF-dependent transcriptional activity in colon cancer [202] and heavily affected Wnt signaling by modulating peculiar miRNAs in GBM [203]. Wnt-dependent transcription was also blocked by the green tea-derived epigallocatechin-3-gallate (EGCG) through induction of peculiar transcriptional repressors [204] or by preventing β-catenin nuclear translocation [205]. Certain indirubin derivatives such as 6-bromoindirubin-oxime (BIO), -acetoxime (BIA), and indirubin-3′-oxime (IO) demonstrated a strong anti-invasive effect in GBM models through inhibition of GSK-3 [206]. Moreover, some derivatives from natural sources, including naringenin (NAR) and phloroglucinol (PGL) have been suggested to antagonize canonical Wnt signaling receptors [207].

However, due to a general lack of specificity and poor knowledge of their molecular mechanisms of action, many of these natural products have failed to proceed to the clinal phases of experimentation, with only a few of them, such as vitamins and resveratrol, being included in clinical trials, essentially based on their pro-differentiation activity on various cancers [28].

### 5.3. Small Molecule Inhibitors

Several small chemical inhibitors of the Wnt/β-catenin signaling have been identified/developed during recent years through in silico and in vitro high throughput screening (HTS) approaches. Nevertheless, before providing a representative description of these discovery process-derived compounds, at least a few drugs, characterized by a repurposed action against Wnt signaling and a reported anti-cancer effect in multiple GBM models, should be described. The anthelmintic compound niclosamide, in addition to a prominent anti-migratory action on human cells [208,209], displayed a consistent inhibitory effect on nuclear β-catenin accumulation, and was therapeutically effective in in vivo GBM models [208,277]. Similarly, another anthelminthic compound, pyrvinium pamoate, was demonstrated to significantly reduce self-renewal and proliferation of GSCs, in part through inhibition of the Wnt/β-catenin transcriptional activity [210], although this may represent only a secondary output, dependent on the modulation of other pathways [278]. Moreover, the antipsychotic drug quetiapine has been recently reported to induce oligodendroglial differentiation of GSCs through the reduction of GSK-3β phosphorylation [211]. As a final example, pioglitazone, an antidiabetic drug used to lower blood glucose levels in type 2 diabetes patients, was also shown to reduce β-catenin expression in patient-derived GBM cultures [212].

In addition to repurposed drugs, several other compounds with the ability to interfere with various Wnt pathway components have been identified and developed during the last 20 years. A brief description of these compounds, subgrouped according to their suggested mechanism of action, is reported here below.

#### 5.3.1. Antagonists of β-Catenin/Transcriptional Co-Activators Interaction

A small series of Wnt signaling inhibitors, acting as β-catenin/TCF interaction disrupting agents, was first identified in 2004 by Lepourcelet et al. through a HTS approach of a large library of compounds [279]. However, these compounds were not further developed due to lack of selectivity. Then, the synthetic compound PNU 74654 was identified for its ability to antagonize β-catenin/TCF-4 binding [216], with recently reported biological activity against both colorectal and hepatocellular carcinomas [213,217], but not brain tumors. Through a similar strategy, 2,4-diamino-quinazoline was also identified as a β-catenin/TCF-4 inhibitor, with therapeutic effects in colorectal and gastric cancers [214,215].

ICG-001 exerts its Wnt signaling inhibitory properties by selectively binding the β-catenin transcriptional co-factor CBP, but not its homologous p300, thus only suppressing a CBP-dependent transcriptional program [219], with reported pro-differentiation activity against GSCs [221]. More recently, the ICG-001 derivative PRI-724 was developed as a second-generation β-catenin/CBP antagonist. PRI-724 is endowed with increased potency and selectivity [280], thus entering different phase I/II clinical trials for the treatment of both leukemia and solid tumors, thanks to its verified safety in preclinical studies [222]. Furthermore, PMED-1 was found to weaken the β-catenin/CBP interaction but with poorer activity, despite its highly structural homology with ICG-001 [218]. E7386 is considered the first orally available compound of this class of inhibitors, however little is known about its precise mechanism of action and relative biological activity [222]. Interestingly, ICG-001 was shown to interact with the β-catenin-associating portion of the CBP N-terminus, which also contains retinoic acid and vitamin-D-interacting sites, suggesting that these compounds may share identical mechanisms of action [28,220].

The more recent inhibitors of the β-catenin-containing transcriptional complex [13] are BC2059 and methyl 3-([(4-methylphenyl)sulfonyl]amino)benzoate (MSAB), which attenuate active β-catenin levels, eventually impacting on its transcriptional activity [221,223,224]; CGP049090, CWP232228, and LF3, all inhibiting the β-catenin/TCF4 complex by sabotaging their interaction [225,226,227]; and SAH-BCL9, developed to block the interaction of β-catenin with B cell lymphoma 9 (BCL9), a co-activator of β-catenin-mediated transcription, by directly interacting with β-catenin and dissociating the β-catenin/BCL9 complexes [228].

#### 5.3.2. DVL Inhibitors

This class of compounds is designed to inhibit the capability of DVL to intracellularly transduce Wnt ligand-dependent FZD receptor activation. NSC668036, FJ9, and 3289-8625 have been reported to interact with the PZD protein–protein interaction domain of DVL and to block Wnt signaling activation in vivo in Xenopus embryos and lung cancer mouse models [229,230,231].

#### 5.3.3. Axin Modulators

One of the first examples of compounds affecting Axin protein stability were the Intracellular Wnt Response (IWR) inhibitors. IWRs are targeted at blocking Axin destruction, thus favoring the suppression of Wnt signaling through the increase of β-catenin proteasomal degradation [232,234]. Comparable results, through Axin stabilization, were achieved by using SEN46 and the Tankyrase inhibitor XAV939 in GBM cells [233]. Tankyrase enzymes, belonging to the Poly(ADP-Ribose) Polymerase (PARP) family of transferases, are known to promote ubiquitin-dependent degradation of Axin proteins, thus enhancing Wnt signaling activation. Tankyrase inhibitors stabilize Axins, critically affecting Wnt pathway components [281]. XAV939 has been widely used for experimentally treating GBM models, displaying promising chemo- and radio-sensitizing effects [136,236]. Additional Tankyrase inhibitors, such as AZ1366, G007-LK, and NVP-TNKS656, have been used with similar therapeutic effects in several cancer types, with some of them also displaying efficacy in gliomas [235,237,238,239]. Despite being characterized by a promising Wnt signaling inhibitory action, since Tankyrase targeting may exert additional undesired effects on multiple intracellular pathways, such inhibitors have failed to proceed toward clinical investigation.

#### 5.3.4. Inhibitors of Wnt Ligands Production (PORCN Inhibitors)

The HTS approach implemented by Chen and colleagues in 2009, besides identifying IWRs, uncovered a subset of Inhibitors of Wnt Production (IWPs) [232]. Wnt signaling inhibition mediated by these compounds is based on their ability to strongly reduce the levels of lipidated Wnt ligands by antagonizing O-acyltransferase Porcupine (PORCN) [232,282]. Indeed PORCN-dependent acylation of Wnt ligands eases their lipidation, favoring their secretion, the generation of proper extracellular ligand gradients, and, finally, their biological activity [282,283]. LGK974 was reported to hamper Wnt signaling through PORCN inhibition in several solid cancers, including both mammary and brain tumors, without displaying toxic effects [94,133,242,244]. ETC-159, WNT-C59, and GNF-6231 were identified, through various screening approaches and progressive chemical modifications, as more potent PORCN inhibitors than LGK974, able to dramatically inhibit tumor growth in colorectal, nasopharyngeal, and breast cancer models, respectively [240,241,243]. As for LGK974, no toxic effects of these compounds have been recognized. Interestingly, LGK974 was the first PORCN inhibitor to enter a clinical trial for the treatment of various solid cancers generally characterized by the over-activation of Wnt signaling [284]. Therefore, inhibitors of PORCN can be considered effective therapeutics against several cancers with known Wnt pathway over-activation. 

#### 5.3.5. Others

ROR receptors may participate in Wnt signaling by serving as co-receptors for FZDs and enhancing specific Wnt-5a/ROR/FDZ non-canonical intracellular signaling (Figure 1), which is involved in tumor cell proliferation and invasion/metastasis, particularly in bones [285,286,287]. KAN 0439834 is a specific small molecule ROR1 inhibitor which has been reported to retain cytotoxic effects against ROR1-expressing cancer cells [245]. In addition, ROR1 has been used as a promising target for the development of antibodies for Wnt signaling inhibition (see also the following paragraph).

Another promising agent is ONC201, generally indicated as a dopamine receptor D2 antagonist, which has been shown to reduce the expression of several Wnt pathway components, including peculiar Wnt ligands, receptors, and co-factors [247]. In addition, besides its potential effects against the Wnt signal, ONC201 is emerging as a promising TNF-related apoptosis-inducing ligand (TRAIL)-inducing compound in GBM, with reported effectiveness in preliminary small patient cohorts [288,289].

We and others previously suggested that certain HDAC inhibitors of the hydroxamate class, including suberoylanilide hydroxamic acid (SAHA, Vorinostat), could be indicated as Wnt signaling antagonists since they were able to deplete TCF4-dependent Wnt pathway activation in colon carcinoma cells [246] and to cause a general shutdown of the Wnt signaling cascade in GBM cells, finally impairing their proliferation and migration [248]. In the same context, additional epigenetic modulators such as demethylating agents (i.e., azacytidine) have been proposed to hamper Wnt signaling activation by inducing the re-expression of several Wnt pathway inhibitor genes, which are generally turned off due to promoter hypermethylation in gliomas and other tumors, as already stated in previous paragraphs [29,101,106,107,108,249,250].

Finally, small molecule inhibitors such as proteolysis-targeting chimeras (PROTACs) have recently attracted interest for their potential application against several cancers by targeting protein degradation. As an example, a novel PROTAC β-catenin has demonstrated efficacy in inhibiting Wnt signaling in colorectal cancer cell lines and patient-derived organoids [251]. These approaches may represent an interesting option for future drug development, although experimental validation is still required for a proper assessment of their efficacy and safety profiles.

### 5.4. Antibodies

Several therapeutic antibodies against Wnt signaling pathway components have been developed in recent years. Indeed, based on the relevance of ROR1 expression in human cancers, cirmtuzumab was developed as a humanized antibody inhibiting Wnt-5a-ROR-induced signaling, which then entered a phase I clinical trial for CLL patients [252]. Along this line, ROR1 CAR-T cells have also been developed, demonstrating high efficacy and safety in preclinical animal models [253]. In addition, antibody–drug conjugates (ADC) have also been recently developed for targeting ROR1, including VLS-101, which comprises a cirmtuzumab-linked anti-microtubule toxin such as monomethyl auristatin E [255], and NBE-002, an anti-ROR1 antibody carrying a novel anthracycline payload [254].

Additional antibodies have been mainly developed to trap Wnt ligands or target FZD receptors. Antibodies against Wnt-1, Wnt-2, Wnt-5a (pAb5a-5), and secreted frizzled receptor protein 2 (SFRP2) have all been demonstrated to induce prominent apoptosis and cell death in several solid tumor models [256,257,258,259,260]. Moreover, chimeric proteins composed of the FZD8 peptide fragments fused with the human FC domains (F8CRDhFc and Ipafricept) have been also designed and tested with promising efficacy and adequate tolerability [261,262]. In the group of antibodies targeting FZD receptors, vantictumab (OMP18R5), which targets several FZDs [263,264], the ^90^Y labeled OTSA101-DPTA-90Y monoclonal antibody (mAb) [267], TT641 polyclonal antibody [266], and MAb 92-13 [265], all designed to target FZD10, have shown promising therapeutic effects against several solid tumors. Moreover, the anti-R-Spondin 3 (RSPO3) mAb OMP-131R10 has demonstrated promising canonical Wnt signaling attenuation, by inducing FZD proteasomal degradation in non-cancerous models [268].

Despite increasingly promising results being reported for the use of these therapeutics against several cancers, we still have no data on the possible implementation of Wnt-targeting antibody-based therapies in the brain tumor context.

## 6. Additional Considerations and Perspectives

Although we provided evidence that emerging compounds targeting Wnt signaling (or its ancillary modulators), may represent an effective therapeutic strategy against several cancers, inhibition of Wnt signaling in brain tumors still remains a challenging deal, due to its recognized role in brain vascularization and blood–brain barrier (BBB) integrity. Indeed, Wnt-7 ligands produced by neural progenitors activate canonical Wnt signaling through FDZ binding, thus stimulating ECs [290,291]. In addition, the Wnt pathway is known to regulate the expression of pro-angiogenic factors such as Vascular Endothelial Growth Factor (VEGF) which, besides serving as a relevant target of anti-angiogenic therapies in several contexts, including GBM, may dramatically affect the normal physiological functions of brain vasculature [292]. Therefore, further studies will be needed in order to understand the impact of Wnt signaling inhibitors on GBM angiogenesis and normal brain microvascular network, since their modification could provoke undesired BBB alterations, with relevant consequences on its permeability to certain drugs.

It is mandatory to also consider the tight epigenetic regulation that acts upstream of the Wnt pathway function. In particular, it has been reported that hundreds of non-coding RNAs are able to regulate (positively or negatively) several Wnt signaling components. Although these have not been mentioned within the previous sections, an exhaustive summary of the main micro-RNAs and long non-coding (lnc)RNAs able to affect Wnt signaling was recently reported by Daisy Precilla et al. [293]. 

Collectively, the factors to examine and the problems to overcome are multiple and could be also related to the mechanism of action of the proposed drugs/therapeutics and the knowledge that Wnt signaling is fundamentally involved in the homeostasis of nearly all adult tissues. Indeed, there are few clinical trials verifying the use of Wnt inhibitors in brain tumors (summarized in Table 2; https://clinicaltrials.gov/, accessed on 21 April 2023), with most of them withdrawn early due to bone and gastrointestinal toxicity [294]. As an example, the great promises of vantictumab were not fulfilled when clinical trials stopped due to bone-related safety [295]. Indeed, the expression of multiple secreted Wnt ligands has been associated with the regulation of bone integrity, density, and mineralization [296].

Based on these considerations, the blockade of the Wnt system, which in normal tissues controls vital functions, is thus limited in its clinical applications. Consequently, it is necessary to identify additional pharmacological strategies to restrict Wnt inhibition exclusively, or mainly, at the level of cancer cells.

One proposed approach could be the specific targeting of defined Wnt branches, rather than the whole Wnt system. Indeed, as described above, the non-canonical signaling downstream of the Wnt/ROR cascade is more associated with cell migration and invasion. For this reason, besides potential concerns regarding its impact on the wound healing process, this pathway may be considered less toxic systemically, but nonetheless endowed with therapeutic efficacy [305]. In agreement with this view, preclinical and clinical trials investigating ROR1 as a drug target are emerging as relevant anti-cancer approaches. Adding further complexity, one may also keep in mind that Wnt ligands, through the interaction with multiple receptors, often result in redundant intracellular responses, sustained by non-shared molecular machineries. This should stimulate the research toward the setup of combined target strategies, with the promise to enhance the possibility to achieve an efficient pharmacological suppression of peculiar Wnt-dependent cellular functions. Accordingly, Wnt signaling inhibition may represent only one tile of a more complex multi-target therapeutic puzzle that, unfortunately, is still far from being solved. Nevertheless, although we provided evidence that a full inhibition of the Wnt-dependent signaling cascades should be viewed with caution due to their pleiotropic action, the recent advances made in the preclinical development of multiple potent Wnt signaling inhibitors should not be discarded a priori, but rather considered a fundamental basis for increasing the feasibility of clinically relevant Wnt inhibition to treat patients. 

## 7. Conclusions

The Wnt pathway is a complex, multifaceted, and tightly regulated signaling pathway involved in vertebrate development and tissue homeostasis. The role of Wnt is also pivotal during adulthood, thus impacting on the functionality and structure of several organs. In this review, we focused on the role of Wnt in determining brain tumor onset, aggressiveness, and microenvironment. Moreover, we evidenced that Wnt signaling also depends on, and modulates, several other pathways to control cell differentiation and sensitization to therapies. In this context, the modulation of the Wnt signal achieved through specific compounds or additional epigenetic mechanisms must be finely tuned for its activation level and correct time of intervention during cancer patient treatment. Despite several clinical trials concentrating on Wnt pathway inhibition as a promising anti-cancer therapy, data collected so far suggest that targeting multiple signaling pathways, aberrantly activated in tumor cells, may be the only reliable strategy to include Wnt signaling inhibitors in the future treatment of multiple cancer types, with a clear anticipated benefit for patients.

## Figures and Tables

**Table 1:** Summary of Wnt signaling inhibitors mentioned within this review.

Class	Agent	Proposed Mechanism/Target	References
NSAIDs	aspirin and indomethacin	reduction of β-catenin/TCFs transcriptional activity	[180,181,182,183,184]
	sulindac	reduction of β-catenin nuclear localization	[185,186,187]
	celecoxib and diclofenac	degradation of TCFs	[46,188,189,190]
Natural compounds	retinoids	increase of Dab-2 and Axin	[191]
	vitamin D	increase of DKK-1 and 4	[192]
	DIF-1 and 3	GSK3-β and cyclin D1	[193,194]
	curcumin and shikonin	β-catenin activation	[195,196,197]
	trichosantin	modulation of LGR5	[198]
	diallyl trisulfide	modulation of LRP6	[199]
	*Rhodiola crenulata* and resveratrol	reduction of β-catenin nuclear localization	[200,201]
	quercetin	destruction of β-catenin/TCFs binding	[202,203]
	EGCG	increase of Wnt transcriptional repressors	[204,205]
	BIO, BIA, and IO	inhibition of GSK-3	[206]
	NAR and PGL	antagonism on Wnt receptors	[207]
Small molecules	niclosamide	reduction of β-catenin nuclear localization	[208,209]
	pyrvinium pamoate	reduction of β-catenin transcriptional activity	[210]
	quetiapine	reduction of GSK-3β phosphorylation	[211]
	pioglitazone	reduction of β-catenin expression	[212]
	PNU 74654 and 2,4-diamino-quinazoline	inhibition of β-catenin/TCF4 binding	[213,214,215,216,217]
	ICG-001, PRI-724 and PMED-1	inhibition of β-catenin/CBP binding	[218,219,220,221]
	E7386	unknown	[222]
	BC2059 and MSAB	reduction of active β-catenin	[221,223,224]
	CGP049090, CWP232228, and LF3	inhibition of β-catenin/TCF4 binding	[225,226,227]
	SAH-BCL9	inhibition of β-catenin/BCL9 binding	[228]
	NSC668036, FJ9, and 3289-8625	inhibition of DVL	[229,230,231]
	IWRs and SEN46	inhibition of Axin destruction	[232,233,234]
	XAV939, AZ1366, G007-LK, and NVP-TNKS656	inhibition of Tankyrase	[136,235,236,237,238,239]
	IWPs, LGK974, ETC-159, WNT-C59, and GNF-62	inhibition of PORCN	[94,133,232,240,241,242,243,244]
	KAN 0439834	inhibition of ROR1	[245]
	ONC201 and SAHA	reduction of multiple Wnt signaling components	[246,247,248]
	azacytidine	increase of Wnt inhibitor expression	[249,250]
	PROTAC β-catenin	induction of β-catenin degradation	[251]
Antibodies	Cirmtuzumab	reduction of Wnt-5a/ROR signaling	[252]
	CAR-T cells	targeting of ROR1	[253]
	VLS-101 and NBE-002	ADCs targeting ROR1	[254,255]
	anti-Wnt-1 mAb	targeting of Wnt-1	[256,257]
	anti-Wnt-2 mAb	targeting of Wnt-2	[258]
	pAb5a-5	targeting of Wnt-5a	[259]
	SFRP2 mAb	targeting of SFRP2	[260]
	F8CRDhFc and Ipafricept	targeting of FZD8	[261,262]
	Vantictumab	targeting multiple FZDs	[263,264]
	OTSA101-DPTA-90Y, TT641 and MAb 92-13	targeting of FZD10	[265,266,267]
	OMP-131R10	targeting of RSPO3	[268]

**Table 2:** Summary of ongoing and recently completed (within 5 years) clinical trials involving the use of previously described Wnt signaling inhibitors in the context of brain tumors. “Active” means a clinical trial that is formally active, but has not yet recruited patients. mut: mutated.

Drug	Brain Tumor	Recruitment Status	Phase	References	
Celecoxib	Low and high-grade gliomas	Completed	I	NCT02115074	
	Glioblastoma	Completed	II	NCT00112502	[271,297]
	Recurrent glioblastoma	Completed	I–II	NCT02770378	[298,299]
	Recurrent MB, EPD and ATRT	Recruiting	II	NCT01356290	[300]
Curcumin	High-grade gliomas	Recruiting	I–II	NCT05768919	
ETC-159	Unresectable refractory solid tumors	Recruiting	I	NCT02521844	
ONC201	Diffuse gliomas	Recruiting	II	NCT05009992	[301]
			III	NCT05580562	
			III	NCT05476939	
	Recurrent H3K27M-mut glioma	Active	II	NCT03295396	
	H3K27M-mut gliomas	Active	I	NCT03416530	[302]
			II	NCT02525692	[303]
	Advanced solid tumors	Completed	I	NCT02250781	[304]
SAHA	Diffuse intrinsic pontine glioma	ActiveCompleted	I	NCT02420613	
			I–II	NCT01189266	
	High-grade glioma	Active	II–III	NCT01236560	
			I	NCT00268385	
	Recurrent glioblastoma	CompletedActive	I–II	NCT01266031	
			I–II	NCT00555399	
	Glioblastoma	CompletedActive	I–II	NCT00731731	
			I	NCT03426891	
	Embryonal tumors of the CNS	Completed	I	NCT00867178	
Azacytidine	Recurrent gliomas (IDH1/2-mut)	Recruiting	II	NCT03666559	
	Gliomas (IDH1-mut)	Completed	I–II	NCT03684811	
	Glioblastoma	Completed	I	NCT02223052	
	Recurrent posterior fossa EPD	Recruiting	I	NCT03572530	
			Early I	NCT04958486	
	Recurrent/refractory pediatric brain tumors	Active	I	NCT03206021	
OMP-131R10	Refractory solid tumors	Completed	I	NCT02482441	

## Data Availability

Not applicable.
